# Assessment of Using Occlusal Splints Without Other Adjunctive Treatment Modules in the Management of Temporomandibular Disorders: A Systematic Review of Literature

**DOI:** 10.7759/cureus.89955

**Published:** 2025-08-13

**Authors:** Mehdi Chahrour, Bachar Reda

**Affiliations:** 1 Orthodontics and Dentofacial Orthopaedics, Lebanese Dental Association, Beirut, LBN; 2 Orthodontics, Lebanese Orthodontic Society, Beirut, LBN; 3 Medical, Surgical, and Health Sciences, University of Trieste, Trieste, ITA

**Keywords:** occlusal splint, oral appliances, orofacial pain, pain management, temporomandibular disorders

## Abstract

Temporomandibular disorders (TMDs) significantly affect an individual's quality of life by limiting jaw mobility, causing chronic pain, and contributing to psychological distress. TMDs are more prevalent among adult populations and are reported more frequently in women than men. The multifactorial etiology of TMDs, including anatomical, physiological, psychological, and functional factors, contributes to the challenges of accurate diagnosis and effective management. Among the various treatment options, occlusal splints are widely used in clinical practice for their potential to alleviate symptoms. These devices are designed to alter the occlusal aspects of the teeth and redistribute jaw forces, aiming to reduce joint loading, minimize muscle tension, and protect against bruxism. However, despite their widespread use, the effectiveness of these treatments as the sole modality for TMDs remains a subject of considerable debate within the dental and medical communities. Therefore, this review aims to evaluate the effectiveness of occlusal splints in managing symptoms of TMDs and to determine their role in improving patient outcomes by synthesizing existing evidence from the literature. It will focus on key outcomes, including pain reduction, improved jaw function, and enhanced quality of life, in addition to the potential risks and benefits associated with their use. Occlusal splints may offer benefits in reducing pain and improving jaw function in the management of TMD, but their effectiveness as a standalone treatment remains uncertain, highlighting the need for individualized, multidisciplinary approaches and further research to define their optimal use.

## Introduction and background

The temporomandibular joint (TMJ) is one of the most complex and frequently used joints in the human body. It is classified as a ginglymoarthrodial joint, combining hinge (ginglymoid) and gliding (arthrodial) movements, which allow the joint to rotate and slide within the parasagittal plane during functional activities or movements [1,2]. This three-dimensional range of movement is essential for activities such as chewing, speaking, and parafunctional behaviors, including lateral bruxing or static clenching of the dentition, as well as facial expressions [3]. Despite its importance, the TMJ can be affected by a range of conditions collectively known as temporomandibular disorders (TMDs). TMDs encompass painful and non-painful conditions affecting the muscles of mastication, the TMJ itself, and contiguous structures [4,5]. Alongside pain, patients frequently present with symptoms such as limited jaw mobility, headache, tinnitus, TMJ sounds (click, crepitus), and disc displacement with or without reduction [6-10].

The multifactorial etiology of TMDs, encompassing anatomical, physiological, psychological, and functional factors, presents challenges for accurate diagnosis and effective management [11]. As such, TMDs can significantly impact an individual's quality of life, causing chronic pain, functional limitations, and psychological distress [7-9,12]. Among the various treatment options, occlusal splints are widely used in clinical practice for their potential to alleviate symptoms. These devices are designed to alter the occlusal aspects of the teeth and redistribute jaw forces, thereby reducing joint loading, minimizing muscle tension, and protecting against bruxism [6,7,13,14]. However, despite their widespread use, the effectiveness of these treatments as a standalone treatment for TMDs remains a subject of considerable debate within the dental and medical communities.

This systematic review evaluates the effectiveness of occlusal splints in managing TMDs by synthesizing evidence from randomized controlled trials (RCTs), cohort studies, and prospective and experimental studies. It examines outcomes such as pain reduction, improved jaw function, and quality of life, alongside the potential risks and benefits associated with their use. The findings will contribute to the body of knowledge and provide evidence-based recommendations to healthcare professionals for better management strategies.

## Review

Materials and methods

A systematic literature search was conducted in PubMed, Cochrane, Web of Science, Scopus, and gray literature databases to identify relevant peer-reviewed studies. Medical Subject Headings (MeSH), developed by experts in the field of orofacial pain, were used during the search. We followed the PRISMA (Preferred Reporting Items for Systematic Reviews and Meta-Analyses) guidelines during all stages.

Search Strategy

A literature search was conducted by one author in two databases for articles published before December 10, 2023. The search strategy included the following MeSH terms: "Occlusal splint", "Temporomandibular joint OR Temporomandibular Disorders", and "Pain OR Pain Management", which were first searched independently. We combined our search results into one search using the Boolean operator AND as follows: ("Occlusal splint") AND ("Temporomandibular joint" OR "Temporomandibular disorders") AND ("Pain" OR "Pain management"), and no limits were applied. Only the free full-text filter was used to refine the search and ensure accessibility.

Inclusion and Exclusion Criteria

Using the PICO framework (Table 1), the inclusion and exclusion criteria were defined as follows: Population: all patients diagnosed with TMDs; Intervention: use of occlusal splint for the management of TMDs; Comparison: comparators included a range of treatment options from no treatment to alternatives, including exercises, injections, or surgical procedures; Outcomes included symptom reduction, such as pain relief and improved jaw function, with quality of life being a secondary outcome. We excluded studies with multiple outcomes and unrelated interventions, publications not available in the English language, and studies without full-text access. Studies with similar interventions were grouped together for synthesis.

**Table 1:** PICO framework PICO: population, intervention, comparison, outcome; TMD: temporomandibular disorder

PICO	Inclusion criteria	Exclusion criteria
Population	Any patient diagnosed with TMD	Any patient not diagnosed with TMD
Intervention	Occlusal splint for management of TMD	Studies that do not use an occlusal splint to manage TMD
Comparison	Studies that include a control group, such as no treatment, a placebo, or other interventions	Studies whose comparisons are not related to the management of TMDs. For example, studies focusing on comorbidities
Outcomes	Reduction in pain, improvement in jaw mobility or jaw function, and improvement in quality of life	Studies that do not have any of the outcomes of interest. For example, studies related to costs

Risk of Bias Assessment

The study authors independently evaluated the risk of bias using the Revised Cochrane Risk-of-Bias tool for randomized trials (RoB 2) [15]. Each study's overall risk of bias was classified as low risk if all domains were rated low risk; some concerns if at least one domain raised concerns, but no domain was rated high risk; and high risk if one or more domains were rated high risk or if multiple domains raised concerns that substantially reduced confidence in the results. The assessment included five key domains: randomization process, deviations from intended interventions, missing outcome data, measurement of the outcome, and selection of the reported results.

For non-randomized studies, the Modified Newcastle-Ottawa Scale (NOS) was used to assess risk of bias [16]. This scale evaluates study quality based on the number of stars awarded in three domains: Selection, Comparability, and Outcome/Exposure. Studies are classified as good quality when they receive 3 or 4 stars in Selection, 1 or 2 stars in Comparability, and 2 or 3 stars in Outcome/Exposure; fair quality when they receive 2 stars in Selection, 1 or 2 stars in Comparability, and 2 or 3 stars in Outcome/Exposure; and poor quality when they receive 0 or 1 star in Selection, 0 stars in Comparability, or 0 or 1 star in Outcome/Exposure. Refer to Supplementary file 1 in the Appendices for detailed scoring.

The included case series study was assessed using the Joanna Briggs Institute (JBI) Critical Appraisal Checklist for Case Series [17]. Detailed scoring is presented in Supplementary file 2.

Results

Study Selection Process

**Figure 1:**
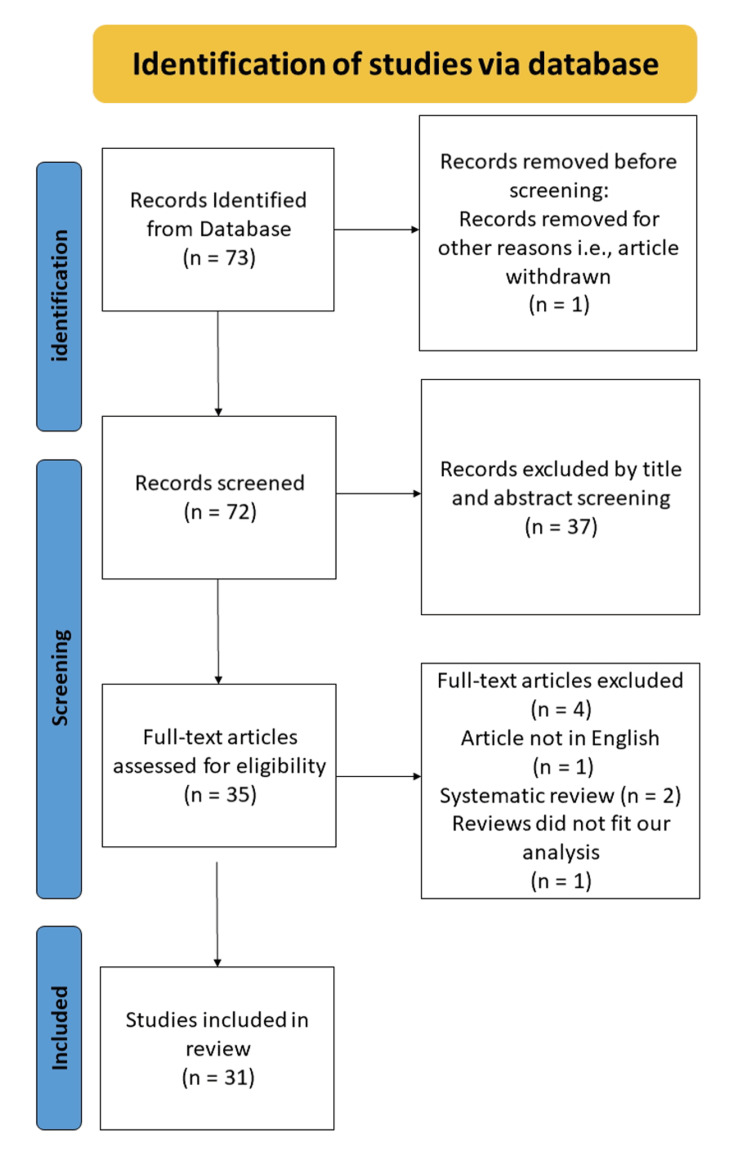
PRISMA flow diagram PRISMA: Preferred Reporting Items for Systematic Reviews and Meta-Analyses

From the initial search, which was completed on December 10, 2023, a total of 634 articles were identified and screened by their titles. To ensure accessibility, we filtered for free full-text articles, resulting in 73 articles (Cochrane (n=43) and PubMed (n=30)) with no duplicate records. One record was removed prior to the screening stage, as it had been withdrawn from the journal in which it was originally published. No automation tool was used in this process. The titles and abstracts of the remaining 72 records were screened independently by two reviewers, and discrepancies were resolved through discussion with a third reviewer who specializes in the field of research to ensure they focused on the objectives of the review. Consequently, 35 articles were included. A final review of the full papers was also conducted by one author, and four papers were removed because one was not in the English language, two were systematic reviews, and the other paper did not fit our objectives. Ultimately, 31 articles were included in this systematic review. Figure 1 summarizes the study selection process presented using a PRISMA flow diagram in accordance with the PRISMA guidelines.

Study Characteristics

The studies included in this review were case series, retrospective studies, and RCTs with varying designs, including single-blind, double-blind, or unblinded. The interventions assessed across the studies ranged from primary or conservative treatments, including exercises, therapy, counseling, and medications, to more advanced approaches, like laser therapy, botulinum toxin infiltration, and surgical procedures. To align with our study aim, which focuses on occlusal splints, we also included studies evaluating different types of occlusal splints, including those with soft, hard, and non-occluding designs. The selected studies covered both population groups (males and females) across various age groups. We did not find any study that included children in their sample. Table 2 presents a detailed summary of the characteristics of the included studies.

**Table 2:** Characteristics of the included studies RCT: randomized controlled trial; TMD: temporomandibular disorder; TMJ: temporomandibular joint; NSAID: nonsteroidal anti-inflammatory drug; DDWR: disc displacement with reduction; TENS: transcutaneous electric nerve stimulation

No.	Author/study	Study design	Study population	Intervention	Treatment duration/evaluation
1	Madani et al., 2023 [8]. Comparative efficacy of different thicknesses of soft and hard splints in reducing clinical symptoms in patients with temporomandibular disorders.	RCT	60 patients randomized into 4 groups. 46 females and 14 males.	Patients randomized into occlusal splints: soft, hard, 1 mm thick, and 3 mm thick	7,30, and 90 days.
2	Hara et al., 2013 [10]. A novel vibratory stimulation-based occlusal splint for alleviation of TMD painful symptoms: a pilot study.	Non-randomized crossover study	10 patients who used stabilization splints for 2 months prior to the study: 3 males and 7 females	Active Vibos vs. inactive Vibos: During sleep time only, patients utilized the active and inactive Vibos for 15 consecutive days	15 days.
3	Alvarez-Arenal et al., 2002 [9]. Effect of occlusal splint and transcutaneous electric nerve stimulation on the signs and symptoms of TMD in patients with bruxism.	A crossed-design experimental study	24 patients: 15 males and 9 females. The average patient’s age was 36.5.	Occlusal splint vs. TENS: Joint and muscle palpation for each patient. Each patient also wore occlusal splints 24 hours a day except during meals for a period of 45 days.	45 days.
4	Conti et al., 2006 [13]. The treatment of painful temporomandibular joint clicking with oral splints A randomized clinical trial.	RCT double-blinded.	57 patients with signs of disk replacement and TMJ pain.	Patients randomized to 3 groups: bilateral balanced splint, canine guidance splint, and non-occluded splint.	15 days, 1 month, 3 months, and 6 months.
5	Wassell et al., 2004 [18]. Treatment of temporomandibular disorders by stabilizing splints in general dental practice: results after initial treatment.	Unilateral crossover design/not blinded	78 patients: 69 females and 9 males.	Patients randomized to 2 groups: stabilizing splint; non-occluding splint.	6 weeks, 3 months.
6	Mazzeto et al., 2009 [14]. Analysis of TMJ Vibration Sounds Before and After Use of Two Types of Occlusal Splints.	RCT	31 patients with TMD: 29 females and 2 males.	Patients divided into 2 groups: stabilizing splint; anterior repositioning splint by means of the electro-vibratography (EVG).	4 weeks.
7	El-Shaheed et al., 2023 [19]. Efficacy of stabilization splint and low-level laser therapy for patients with chronic closed lock from non-reducible displaced temporo-mandibular joint discs: A parallel randomized clinical trial.	RCT	42 patients with chronic closed lock: 35 females and 7 males.	Patients were divided into 3 groups: stabilizing splint; low level laser; and stabilizing splint with low-level laser.	Evaluation done at 1, 2, and 4 weeks and 3 and 6 months.
8	Davis et al., 1997 [20]. The pattern of splint usage in the management of two common temporomandibular disorders. Part III: long-term follow-up in the management of disc displacement with reduction and pain dysfunction syndrome.	Prospective longitudinal cohort	Two groups of patients: 107 in the DDWR group; 48 patients with pain dysfunction syndrome.	Patients divided into 2 groups: anterior repositioning splint for the DDWR group; stabilizing splint for the PDS group.	After 3 months of splint usage/long-term after 3 years of successful therapy.
9	Pihut et al., 2018 [21]. The efficiency of anterior repositioning splints in the management of pain related to temporomandibular joint disc displacement with reduction.	RCT	112 patients with TMDs: 83 females and 29 males.	Patients randomly assigned to either an anterior repositioning splint on the lower arch for 20 hours for 4 months; a biostimulation laser over 12 sessions on the TMJ with the mouth open and while performing self-exercise.	Evaluation at 4 weeks and 16 weeks.
10	Alajbeg et al., 2020 [22]. Effect of occlusal splint on oxidative stress markers and psychological aspects of chronic temporomandibular pain: a randomized controlled trial.	RCT	34 chronic TMD patients.	Patients randomly assigned to 2 groups: stabilizing splint: a hard, flat splint on the upper arch worn at night; placebo splint: 0.5 mm thick, worn at night.	Evaluated at 3 and 6 months.
11	Noguchi et al., 2019 [23]. The effectiveness of stabilization appliance therapy among patients with myalgia.	Case series	62 patients with TMD symptoms: 12 males, 50 females.	Stabilization splint.	2 months.
12	Christidis et al., 2014 [24]. Effectiveness of a Prefabricated Occlusal Appliance in Patients with Temporomandibular Joint Pain: A Randomized Controlled Multicenter Study.	RCT multicenter	48 patients with TMJ: 45 females and 3 males.	Patients randomized into two groups: prefabricated appliance; stabilization splint.	10 weeks.
13	Chang et al., 2010 [25]. Treatment effects of maxillary flat occlusal splints for painful clicking of the temporomandibular joint.	Retrospective cohort	109 patients with unilateral clicking concurrent with preauricular area pain for at least 2 months.	Two groups of treatment options: maxillary flat occlusal splint; no treatment.	90 days.
14	Dalewski et al., 2019 [26]. Comparison of early effectiveness of three different intervention methods in patients with chronic orofacial pain: a randomized, controlled clinical trial.	RCT	90 patients with myofascial pain (mostly women).	Patients randomized into three groups: occlusal splint with NSAID; occlusal splint with dry needling; occlusal splint (control group).	3 weeks.
15	Diraçoğlu et al., 2009 [27]. Arthrocentesis versus nonsurgical methods in the treatment of temporomandibular disc displacement without reduction.	Single-blinded prospective study	120 patients with disk displacement without reduction: 104 females and 16 males.	Patients were given either of the two treatment options: arthrocentesis; combination of splint, hot pack and botulinum.	Patients evaluated at 1, 3, and 6 months.
16	Pihut et al., 2017 [28]. Evaluation of articular disc loading in TMJs after prosthetic and pharmacological treatment in model studies.	RCT	10 patients suffering from TMJ dysfunction.	Patients randomized into two groups: occlusal splint; intramuscular injection of botulinum.	Patients evaluated after 10 days and 14 and 22 weeks.
17	Wänman et al., 2020 [7]. Treatment outcome of supervised exercise, home exercise and bite splint therapy, respectively, in patients with symptomatic disc displacement with reduction: A randomized clinical trial.	RCT	63 females and 27 males.	Patients were randomized into three groups: bite split; unsupervised home exercise; supervised exercise at the clinic.	3 months.
18	Niemelä et al., 2012 [29]. Efficacy of stabilization splint treatment on temporomandibular disorders.	RCT	80 patients with TMDs: 18 males and 62 females.	Patients randomized into two treatment groups: stabilization splint with counseling and exercise; counseling and exercise.	1 month.
19	Sato et al., 1995 [30]. Management of nonreducing temporomandibular joint disk displacement – Evaluation of three treatments.	RCT	77 patients with anterior disk displacement.	Patients divided into three groups: natural course; stabilizing splint; surgery.	Patients evaluated at baseline and 12 months after treatment.
20	Priyadarshini et al., 2021 [31]. Evaluation of prolotherapy in comparison with occlusal splints in treating internal derangement of the temporomandibular joint - a randomized controlled trial.	RCT	34 patients with TMJ internal derangement stages II or III.	Patients divided into 2 groups: phototherapy; splint.	Patients evaluated at 1, 3, 6, and 12 months.
21	Sousa et al., 2020 [32]. Different treatments in patients with temporomandibular joint disorders: a comparative randomized study.	RCT	80 patients with TMDs.	Patients randomly distributed into 4 treatment groups: bite splint; bite splint with betamethasone; bite splint with sodium hyaluronate; bite splint with platelet-rich plasma.	Patients evaluated at baseline, 1 week, 1 month and 6 months.
22	Conti et al., 2015 [33]. Management of painful temporomandibular joint clicking with different intraoral devices and counselling: a controlled study.	RCT	60 patients with disc displacement with reduction: 2 males, 58 females.	Patients randomly divided into three groups: counseling and anterior repositioning splint; counseling and nociceptive trigeminal inhibition clenching suppression system devices; counseling and self-care.	Follow-ups at 2 weeks, 3 weeks, and 6 months.
23	Wahlund et al., 2020 [34]. The course of pain intensity and frequency of adolescents treated because of temporomandibular disorders: A long-term follow-up.	Prospective longitudinal cohort	186 Patients with TMDs: First trial: 122 patients, second trial: 64 patients.	First trial patients were randomly allocated to either information only, information only with an occlusal splint, or information with relaxation therapy.	3 months and at 6 months after treatment.
24	Chen et al., 2022 [35]. Effects of occlusal splint and exercise therapy, respectively, for the painful temporomandibular disorder in patients seeking for orthodontic treatment: a retrospective study.	Retrospective cohort	87 TMD patients: 41 males, 46 females.	Two treatment options: hard stabilization splint; counseling and exercise.	First phase: 4 months.
25	Özkan et al., 2011 [36]. Trigger point injection therapy in the management of myofascial temporomandibular pain.	RCT	50 patients with myofascial TMDs: 44 females, 6 males.	Patients randomized into two groups; stabilization splint (SS); trigger point injection with SS.	12 weeks.
26	Madani et al., 2011 [6]. Comparison of three treatment options for painful temporomandibular joint clicking.	RCT	60 patients with painful TMJ clicking.	Patients randomly divided into anterior repositioning splint therapy; physiotherapy; physiotherapy with splinting.	Group 1: 4 months, Group 2: 4 weeks, Group 3: 5 months.
27	Gupta et al., 2022 [37]. Effectiveness of Vitamin D along with Splint therapy in the Vit D deficient patients with Temporomandibular disorder-a randomized, double-blind, placebo-controlled clinical trial.	RCT	36 patients with TMDs.	Patients randomized into two groups: stabilization splint with vitamin D supplements; stabilization splint with placebo.	3 months.
28	De Vocht et al., 2013 [38]. A pilot study of a chiropractic intervention for management of chronic myofascial temporomandibular disorder.	RCT	80 patients with chronic myofascial TMDs: 64 females, 16 males.	Patients assigned into 4 groups (all with self-care): reversible interocclusal splint; Activator Method Chiropractic Technique (AMCT); SHAM AMCT; self-care only.	6 months.
29	Minakuchi et al., 2001 [39]. Randomized controlled evaluation of non-surgical treatments for temporomandibular joint anterior disk displacement without reduction.	RCT	69 patients with ADDWoR[BR1]	Patients randomly assigned to 3 groups: a control group; self-care with NSAIDs; splint with self-care and NSAIDs.	8 weeks.
30	Wright et al., 1995 [40]. A randomized clinical trial of intraoral soft splints and palliative treatment for masticatory muscle pain.	RCT	30 patients suffering from masticatory muscle pain.	Patients were randomly assigned to the following: soft splint; palliative treatment; no treatment.	Evaluated at 4 and 11 weeks.
31	Mejersjö et al., 2008 [41]. Diclofenac sodium and occlusal splint therapy in TMJ osteoarthritis: a randomized controlled trial.	RCT	29 patients with TMJ osteoarthritis.	Patients randomized into two groups: diclofenac; occlusal splint.	3 months

Risk of Bias in the Studies

A total of 26 RCTs were evaluated using the Revised Cochrane RoB2 tool. Of these, six studies were rated as having a low overall risk of bias, 14 studies were judged to have some concerns in at least one domain without meeting criteria for high risk, and the remaining six studies were rated as having a high risk of bias, primarily due to methodological limitations related to the randomization process, deviations from intended interventions, or outcome measurement. Detailed assessment is presented in Table 3.

**Table 3:** Risk of bias assessment for randomized controlled trials using the RoB2 tool RoB2: Revised Cochrane Risk-of-Bias

Study	Randomization process	Deviations from intended interventions	Missing outcome data	Measurement of the outcome	Selection of the reported result	Overall risk-of-bias judgment
Madani et al. (2023) [8]	Some concerns	Some concerns	Low risk	Low risk	Low risk	Some concerns
Hara et al. (2013) [10]	Low risk	Low risk	Low risk	Some concerns	Low risk	Some concerns
Álvarez-Arenal et al. (2002) [9]	Some concerns	Some concerns	Some concerns	Low risk	Low risk	High risk
Conti et al. (2006) [13]	Low risk	Some concerns	Low risk	Low risk	Low risk	Some concerns
Wassel et al. (2004) [18]	Some concerns	Low risk	Some concerns	Low risk	Low risk	Some concerns
Mazzetto et al. (2009) [14]	High risk	Some concerns	Low risk	Some concerns	Some concerns	High risk
El-Shaheed et al. (2023) [19]	Low risk	Low risk	Low risk	Low risk	Low risk	Low risk
Pihut et al. (2018) [21]	Some concerns	High risk	Low risk	High risk	Some concerns	High risk
Alajbeg et al. (2020) [22]	Low risk	Some concerns	Low risk	Some concerns	Low risk	Some concerns
Christidis et al. (2014) [24]	Low risk	Low risk	Low risk	Low risk	Low risk	Low risk
Dalewski et al. (2019) [26]	Low risk	Some concerns	Low risk	Some concerns	Low risk	Some concerns
Diraçoğlu et al. (2009) [27]	Low risk	Some concerns	Low risk	High risk	Low risk	High risk
Pihut et al. (2017) [28]	High risk	Low risk	Low risk	Some concerns	Low risk	High risk
Wänman et al. (2020) [7]	Low risk	Low risk	Low risk	Low risk	Low risk	Low risk
Niemella et al. (2012) [29]	Low risk	Low risk	Low risk	Low risk	Low risk	Low risk
Sato et al. (1995) [30]	Some concerns	Some concerns	Low risk	Some concerns	Low risk	Some concerns
Priyadarshini et al. (2021) [31]	Some concerns	Low risk	Low risk	Some concerns	Low risk	Some concerns
Sousa et al. (2020) [32]	Some concerns	Low risk	Low risk	Some concerns	Low risk	Some concerns
Conti et al. (2015) [33]	Some concerns	Some concerns	Low risk	Some concerns	Low risk	Some concerns
Özkan et al. (2011) [36]	Low risk	Low risk	Low risk	Low risk	Low risk	Low risk
Madani et al. (2011) [6]	Some concerns	Some concerns	Some concerns	Low risk	Low risk	Some concerns
Gupta et al. (2022) [37]	Low risk	Low risk	Low risk	Low risk	Low risk	Low risk
DeVocht et al. (2013) [38]	Low risk	Some concerns	Some concerns	Some concerns	Low risk	Some concerns
Minakuchi et al. (2000) [39]	Some concerns	Some concerns	Low risk	Low risk	Low risk	Some concerns
Wright et al. (1995) [40]	High risk	High risk	Low risk	High risk	Some concerns	High risk
Mejersjö et al. (2008) [41]	Some concerns	Some concerns	Low risk	Some concerns	Low risk	Some concerns

Four non-randomized studies were assessed using the Modified NOS. Among them, two studies were rated as good quality, while two were considered poor quality, mainly due to limited comparability and inadequate selection criteria. The NOS-based evaluations for these studies are summarized in Table 4. The included case series was appraised using the JBI Critical Appraisal Checklist for Case Series. It clearly defined the inclusion criteria, applied standardized outcome measures (e.g., the Visual Analog Scale), and utilized valid diagnostic procedures performed by trained clinicians. The study also provided detailed clinical and demographic data and reported both objective and subjective outcomes clearly. While the study site and statistical methods were adequately described, it was unclear whether participants were recruited consecutively, and incomplete reporting of exclusions raises potential concerns about selection bias.

**Table 4:** Risk of bias assessment for non-randomized studies using the Modified Newcastle–Ottawa Scale (NOS)

Study	Selection	Comparability	Outcome	Overall risk-of-bias judgment
Davies et al., (1997) [20]	Stars awarded: 2	Stars awarded: 0	Stars awarded: 1	Poor quality
Chang et al., (2010) [25]	Stars awarded: 2	Stars awarded: 0	Stars awarded: 2	Poor quality
Wahlund et al., (2020) [34]	Stars awarded: 4	Stars awarded: 1	Stars awarded: 3	Good quality
Chen et al., (2022) [35]	Stars awarded: 3	Stars awarded: 1	Stars awarded: 2	Good quality

Discussion

Effectiveness of Splint Therapy in the Management of TMDs

This systematic review evaluated the diverse applications and potential benefits of occlusal splints in the management of TMDs [6,7,10,13,14,18-20,22-26,37,41]. While they are widely used in clinical practice, inconsistencies remain across studies regarding the effectiveness of splints as the sole treatment modality. Stabilization splints have consistently shown promise in reducing pain and improving jaw function, particularly with prolonged use. Splint variations in material type, design, and treatment duration introduced differences across studies. Superior outcomes were observed in anterior repositioning splints when used for over four weeks and hard splints with greater thickness (e.g., 3 mm), likely due to their ability to stabilize and redistribute load, underscoring the importance of treatment duration and splint type. This highlights the need for individualized treatment plans to achieve optimal care, as soft splints are more effective in patients with mild dysfunction.

Combination therapies [18,29,35,40,41], involving splints and other treatments such as low-level laser therapy (LLLT), vitamin D, physiotherapy, or NSAIDs (nonsteroidal anti-inflammatory drugs), challenged the role of occlusal splints as a primary intervention/only intervention without adjunctive treatment modalities, as they provided enhanced symptom relief and improved quality of life. Patients treated with a splint combined with NSAIDs reported improved quality of sleep after three weeks of treatment [41]. These findings suggest multimodal approaches are beneficial and might be better suited for acute symptom relief. For example, in a comparative study, combination with diclofenac provided relief within one week compared to splint therapy, which showed improvement after one month. Future research should aim to refine protocols, explore combination therapies, and optimize patient outcomes in clinical practice.

Challenges and Limitations of Splint Therapy

Long-term use of occlusal splints has been shown to improve outcomes; however, their long-term use has the potential of altering the condyle position [37], particularly if they are not precisely designed and/or monitored, especially the hard splint with more thickness. Occlusal splints are also an irreversible treatment option and therefore require careful consideration. Their application universally poses as a limitation, as some studies have reported limited benefits with prefabricated appliances [19]. More robust research is therefore needed to look more into these challenges and limitations.

Alternative Therapies to TMD Management

Exercises, injections, counseling, therapy, and self-care [8,9,27,28,30-36,38,39] are some of the adjunctive therapies used in the management of TMDs, though they demonstrated different degrees of efficacy. Notably, supervised exercise programs were more effective than unsupervised ones, particularly in improving jaw mobility and reducing clicking sounds. In most clinical implementations and research, these alternative therapies have been used alongside splints, and their efficacy when used alone is still a question of interest. Therefore, this calls for further exploration of independent treatment options in managing TMDs.

Surgical Interventions

Generally, surgical methods have been considered as the last resort for unresponsive patients. Patients who underwent surgery after failing non-surgical procedures showed higher success rates compared to those managed using splints or natural remedies [42,43]. This finding reaffirms the stepwise management approach to TMDs where conservative therapies have been exhausted.

Implications for Clinical Practice

The variations in the efficiency of splints highlight the significance of multimodal and stepwise approaches to TMD management. While effective for many patients, healthcare providers should carefully consider patient characteristics such as age, duration, and severity of symptoms when tailoring treatment plans; therefore, these treatments should not be viewed as a universal solution. Considering their limitations, especially in prolonged use, and their irreversibility, care must be taken.

Study limitations

The methodological quality of the included studies was not conducted, which could potentially overestimate or underestimate our findings. Furthermore, few studies have assessed alternative therapies as the only treatment modalities, making direct comparisons with occlusal splints relatively difficult.

## Conclusions

Occlusal splints have demonstrated potential benefits in managing TMDs, particularly in reducing pain and improving jaw function. However, the evidence is mixed regarding their effectiveness as the only treatment without adjunctive treatment modalities. While some studies show positive outcomes, especially with prolonged use and certain types of splints, others suggest limited benefits, particularly when not combined with other therapeutic approaches. The variability in results highlights the need for individualized treatment plans that take into account the specific type of TMD, patient characteristics, and adjunctive therapies. Ultimately, occlusal splints may be a valuable component of a comprehensive, multidisciplinary management strategy for TMDs; however, further research is needed to establish clear guidelines for their optimal use.

## Appendices

Modified Newcastle-Ottawa Quality Assessment Scale

Cohort Studies

Note: A study can be awarded a maximum of one star for each numbered item within the Selection and Outcome categories. A maximum of two stars can be given for Comparability.

**Table 5:** Supplementary file 1

No.	Criterion	Decision rule	Score (*=1, no*=0)	Location in text
Selection				
1	Representativeness of the exposed cohort	Consecutive eligible participants were selected, participants were randomly selected, or all participants were invited to participate from the source population* Not satisfying requirements in part (a), or not stated.		
2	Selection of the non-exposed cohort	Selected from the same source population* Selected from a different source population No description		
3	Ascertainment of exposure	a) Structured injury data (e.g., record completed by medical staff)* b) Structured interview* Written self-report No description		
4	Demonstration that outcome of interest was not present at the start of the study	Yes* No or not explicitly stated		
Comparability				
1	Comparability of cohorts on the basis of the design or analysis	Study controls for previous injury* Study controls for age* Note: Exposed and non-exposed individuals must be matched in the design and/or confounders must be adjusted for in the analysis. Alone statements of no differences between groups or that differences were not statistically significant are not sufficient.		
Outcome				
1	Assessment of outcome	Independent or blind assessment stated, or confirmation of the outcome by reference to secure records (e.g. imaging, structured injury data, etc.)* record linkage (e.g. identified through ICD codes on database records)* Self-report with no reference to original structured injury data or imaging No description		
2	Was the follow-up long enough for outcomes to occur?	Yes (≥3 months)* No (<3 months)		
3	Adequacy of follow-up of cohorts	Complete follow up – all participants accounted for* Subjects lost to follow up unlikely to introduce bias (<15% lost to follow up, or description provided of those lost*) Follow-up rate <85% and no description of those lost provided.) No statement		
		SCORE:		

**Table 6:** Supplementary file 2

	Yes	No	Unclear	Not applicable
Were there clear criteria for inclusion in the case series?	□	□	□	□
Was the condition measured in a standard, reliable way for all participants included in the case series?	□	□	□	□
Were valid methods used for identification of the condition for all participants included in the case series?	□	□	□	□
Did the case series have consecutive inclusion of participants?	□	□	□	□
Did the case series have complete inclusion of participants?	□	□	□	□
Was there clear reporting of the demographics of the participants in the study?	□	□	□	□
Was there clear reporting of clinical information of the participants?	□	□	□	□
Were the outcomes or follow-up results of cases clearly reported?	□	□	□	□
Was there clear reporting of the presenting site(s)/clinic(s) demographic information?	□	□	□	□
Was statistical analysis appropriate?	□	□	□	□
